# Supplementary material for: FastqPuri: high-performance preprocessing of RNA-seq data
Source: BMC Bioinformatics. 2019 May 3;20:226. doi: 10.1186/s12859-019-2799-0 (PMC6500068; doi:10.1186/s12859-019-2799-0)
Supplement: Supplementary file 2 — Archive of FastqPuri. Archive containing all files needed to install and run FastqPuri v1.0.6. Date stamp March 22, 2019. (GZ 47,819 kb) [file 12859_2019_2799_MOESM2_ESM.gz › FastqPuri-1.0.6/html/struct__iparam__trimFilter.html]

FastqPuri: \_iparam\_trimFilter Struct Reference


FastqPuri

Public Attributes |
List of all members

\_iparam\_trimFilter Struct Reference

trimFilter input parameters
More...

`#include <struct_trimFilter.h>`

Collaboration diagram for \_iparam\_trimFilter:

[legend]

|  |  |
| --- | --- |
| Public Attributes | |
| char \* | Ifq |
|  | |
| char \* | Ifq2 |
|  | |
| char \* | Ifa |
|  | |
| char \* | Iidx |
|  | |
| char \* | Iinfo |
|  | |
| char \* | Oprefix |
|  | |
| bool | uncompress |
|  | |
| Adapter | ad |
|  | |
| Bfkmer \* | ptr\_bfkmer |
|  | |
| int | trimQ |
|  | |
| int | trimN |
|  | |
| int | method |
|  | |
| bool | is\_fa |
|  | |
| bool | is\_idx |
|  | |
| bool | is\_adapter |
|  | |
| double | score |
|  | |
| int | minQ |
|  | |
| int | L |
|  | |
| int | minL |
|  | |
| int | nlowQ |
|  | |
| int | kmersize |
|  | |
| int | globleft |
|  | |
| int | globright |
|  | |
| int | percent |
|  | |

## Detailed Description

trimFilter input parameters

## Member Data Documentation

## ◆ ad

Adapter \_iparam\_trimFilter::ad

AdapterDS trimming parameters

## ◆ globleft

int \_iparam\_trimFilter::globleft

number of bases globally trimming from the left

## ◆ globright

int \_iparam\_trimFilter::globright

number of bases globally trimming from the right

## ◆ Ifa

char\* \_iparam\_trimFilter::Ifa

Input fa file (containing contamination sequences)

## ◆ Ifq

char\* \_iparam\_trimFilter::Ifq

Input fq file single stranded

## ◆ Ifq2

char\* \_iparam\_trimFilter::Ifq2

Input fq file read 2

## ◆ Iidx

char\* \_iparam\_trimFilter::Iidx

Input index file (from an input.fa cont file)

## ◆ Iinfo

char\* \_iparam\_trimFilter::Iinfo

Input index info file

## ◆ is\_adapter

bool \_iparam\_trimFilter::is\_adapter

true if filtering adapter sequences

## ◆ is\_fa

bool \_iparam\_trimFilter::is\_fa

true if a fasta file was passed as a parameter

## ◆ is\_idx

bool \_iparam\_trimFilter::is\_idx

true if an index file was passed as a parameter

## ◆ kmersize

int \_iparam\_trimFilter::kmersize

kmersize to look for contamination

## ◆ L

int \_iparam\_trimFilter::L

read length

## ◆ method

int \_iparam\_trimFilter::method

TREE(1), BLOOM(2), 0, when not looking for cont

## ◆ minL

int \_iparam\_trimFilter::minL

minimum read length accepted before discarding a read

## ◆ minQ

int \_iparam\_trimFilter::minQ

minimum quality threshold

## ◆ nlowQ

int \_iparam\_trimFilter::nlowQ

maximum number of lowQ bases accepted before discarding

## ◆ Oprefix

char\* \_iparam\_trimFilter::Oprefix

Output files prefix for single str (PATH/prefix)

## ◆ percent

int \_iparam\_trimFilter::percent

percentage of lowQ bases allowed in a read

## ◆ ptr\_bfkmer

Bfkmer\* \_iparam\_trimFilter::ptr\_bfkmer

bloom filter kmer structure

## ◆ score

double \_iparam\_trimFilter::score

score threshold for matching reads in sequences

## ◆ trimN

int \_iparam\_trimFilter::trimN

NO(0), ALL(1), ENDS(2), STRIP(3)

## ◆ trimQ

int \_iparam\_trimFilter::trimQ

NO(0), FRAC(1), ENDS(2), ENDSFRAC(3), GLOBAL(4)

## ◆ uncompress

bool \_iparam\_trimFilter::uncompress

true if output uncompressed, false otherwise

---

The documentation for this struct was generated from the following file:

- include/struct\_trimFilter.h


---

Generated on Mon Mar 19 2018 23:42:01 for FastqPuri by  

 1.8.14
